# Le cancer en milieu chirurgical pédiatrique au Togo

**DOI:** 10.11604/pamj.2014.17.209.3026

**Published:** 2014-03-16

**Authors:** Komla Gnassingbe, Koffi Mawuse Guedenon, Kokou Kanassoua, Komlan Adabra, Kagnimtassou Kpabi, Gamedzi Komlatse Akakpo-Numado, Gado Napo-Koura, Hubert Tekou

**Affiliations:** 1 Service de Chirurgie Pédiatrique, CHU Sylvanus Olympio, Lomé, Togo; 2 Service de Pédiatrie, CHU Sylvanus Olympio, Lomé, Togo; 3 Service des Urgences Chirurgicales, CHU Sylvanus Olympio, Lomé, Togo; 4 Clinique Médico Chirurgicale, CHU Sylvanus Olympio, Lomé, Togo; 5 Service d'Anatomie Pathologique, CHU Sylvanus Olympio, Lomé, Togo

**Keywords:** Enfant, cancer solide, chirurgie pédiatrique, Togo, child, solid cancer, pediatric surgery, Togo

## Abstract

**Introduction:**

Le but de ce travail était de relever les aspects épidémiologiques des cancers de l'enfant en milieu chirurgical, décrire les problèmes posés par ces cancers et évaluer les résultats de leur prise en charge

**Méthodes:**

Il s'agit d'une étude rétrospective analytique sur dossiers de patients âgés de moins de 15 ans pris en charge dans le service de chirurgie pédiatrique pour cancer solide de preuve anatomopathologique entre janvier 1987 et décembre 2010. Jusqu'en 2010, les hôpitaux publics du Togo ne disposaient pas d'imagerie par résonance magnétique ni de la tomodensitométrie. Il n'existe pas de service d'oncologie pédiatrique, ni de radiothérapie au Togo. Depuis quelques années maintenant, le Togo a intégré le Groupe Franco Africain d'oncologie Pédiatrique (GFAOP) et les patients bénéficient gracieusement des antimitotiques pour la prise en charge de certains cancers.

**Résultats:**

Trente un patients avaient été pris en charge dans le service de chirurgie pédiatrique pour cancer. Parmi eux, il y avait 18 garçons (58,06%) et 15 filles (41,94%). L’âge moyen des patients était de 7,62 ans (extrêmes: 3 mois et 15 ans). Les patients étaient également répartis dans les différentes tranches d’âge. Les circonstances de découverte variaient selon le type de tumeurs. Les tumeurs des tissus mous représentaient 51,61% des cas, les tumeurs germinales 25,81% des cas et les tumeurs osseuses 22,58% des cas. Le délai moyen d’évolution avant la consultation était de 4,6 mois (extrêmes: 2 et 14 mois). Le taux de décès était de 54,84% des cas.

**Conclusion:**

Les cancers solides de l'enfant sont caractérisés par un retard à la consultation et un plateau à visée diagnostique et thérapeutique très limité entrainant de ce fait une forte mortalité.

## Introduction

Le cancer de l'enfant constitue la 2ème cause de mortalité chez l'enfant dans le monde [1]. Il y a une trentaine d'années, dans les pays en développement, 60% des enfants succombaient à leur maladie contre 25% dans les pays développés [2]. Aujourd'hui des avancées significatives enregistrées depuis 40 ans en termes de diagnostic et de traitement permettent de guérir un grand nombre de cancer de l'enfant [3]. Ces avancées n'ont pas encore atteint les pays en voie de développement où le cancer continue de tuer des enfants. En milieu chirurgical, le cancer de l'enfant est peu fréquent et de pronostic mauvais. Cette étude a été réalisée en vue de: relever les aspects épidémiologiques des cancers solides; décrire les problèmes posés par les cancers en milieu chirurgical au Togo; évaluer les résultats de leur prise en charge.

## Méthodes

Il s'agit d'une étude rétrospective analytique sur dossiers de patients âgés de moins de 15 ans pris en charge dans le service de chirurgie pédiatrique pour cancer solide de preuve anatomopathologique entre janvier 1987 et décembre 2010. Les cas de lymphome de Burkitt dans leur localisation abdominale et maxillo faciale dont le traitement était exclusivement médical (chimiothérapie) ont été exclus de notre étude. Le Togo ne dispose jusqu’à ce jour que d'un seul laboratoire d'anatomopathologie avec seulement deux anatomopathologistes; ce laboratoire est basé au CHU de Lomé et reçoit de ce fait les prélèvements de tout le pays. Jusqu'en 2010, les hôpitaux publics du Togo ne disposaient pas d'imagerie par résonance (IRM) magnétique ni de la tomodensitométrie (TDM). Seules deux structures sanitaires privées en disposaient mais les frais n’étaient pas à la portée des populations.

Il n'existe pas de service d'oncologie pédiatrique ni de radiothérapie au Togo. La prise en charge des patients était exclusivement à la charge des parents. Depuis quelques années maintenant, le Togo a intégré le Groupe Franco Africain d'oncologie Pédiatrique (GFAOP) et les patients bénéficient gracieusement des antimitotiques pour la prise en charge des cas de néphroblastome.

## Résultats

**Fréquence et incidence hospitalières:** Durant la période de notre étude, le service d'anatomopathologique a enregistré 7189 cas de cancer dont 752 cas pédiatriques. Parmi eux, 31 ont présenté un cancer solide dont la prise en charge médico chirurgicale a été assurée dans le service de chirurgie pédiatrique. Les cancers solides en milieu chirurgical pédiatrique avaient une incidence de 1 cas par an et une fréquence de 0,4% des cancers diagnostiqués et 4% des cancers de l'enfant au Togo.

**Age des patients:** Selon l’âge, 11 patients (35,48%) avaient moins de 5 ans (Tableau 1) L’âge moyen des patients était de 7,62 ans avec des extrêmes de 3mois et 15 ans.

**Tableau 1:** Répartition des patients selon les tranches d’âge (an)

	Effectif	Pourcentage (%)
< 5	11	35,48
[5-10[	10	32,26
≥ 10	10	32,26
**Total**	31	100,00

**Sexe:** Dix huit patients (58,06%) étaient de sexe masculin contre 13 (41,94%) de sexe masculin. Le sex ratio est de 1,38.

**Délai d’évolution avant la consultation:** Selon le délai d’évolution avant la consultation, les patients étaient repartis de la façon suivante (Tableau 2). Le délai moyen d’évolution était de 4,6 mois avec des extrêmes de 2 et 14 mois.

**Tableau 2:** Répartition des patients selon le délai d’évolution avant la consultation (mois)

	Effectif	Pourcentage (%)
< 3	3	9,67
[3-6[	8	25,81
≥ 6	20	64,52
**Total**	31	100,00

**Circonstances de découverte:** Selon les circonstances de découverte, les patients étaient répartis de la façon suivante (Tableau 3).

**Tableau 3:** Répartition des patients selon les circonstances de découverte

	Effectif	Pourcentage (%)
Masse + pesanteur abdominale	15	48,38
Douleur osseuse + boiterie	12	38,70
Hématurie	4	12,90
Douleur abdominale	3	9,68
Masse cutanée	2	6,45

Un patient pouvait avoir un ou plusieurs signes en rapport avec l'organe malade

**Tissu intéressé:** Le cancer avait intéressé les tissus mous chez 16 patients (51,61%), le tissu osseux chez 13 patients (41,93%) et le tissu germinal chez 2 patients (6,45%).

**Moyen diagnostique:** Tous les patients en dehors du néphroblastome avaient bénéficié d'une biopsie avec examen anatomopathologique qui avait permis de poser le diagnostic. Le diagnostic de néphroblastome a été suspecté à l’échographie puis confirmé après néphrectomie par l'histologie. La Tomodensitométrie et l'Imagerie par résonance magnétique n'ont pas été réalisées chez nos patients.

**Type de cancer:** Selon le type de cancer, les patients étaient répartis de la façon suivante (Tableau 4).

**Tableau 4:** Répartition des patients selon le type de tumeur

		Effectif	Pourcentage (%)
**Tumeur tissus mous**	Néphroblastome	11	51,61
	Hépatome malin cholangiocellulaire	2	
	Angiosarcome	1	
	Adenocarcinome appendiculaire	1	
	Adenocarcinome vésiculo trabéculaire	1	
**Tumeurs germinales**	Tératome malin	5	25,81
	Carcinome embryonnaire	3	
**Tumeurs osseuses**	Sarcome d'Ewing	3	22,58
	Ostéochondrosarcome	4	

**Aspects thérapeutiques:** Selon le type de traitement, les patients étaient répartis de la façon suivante (Tableau 5).

**Tableau 5:** Répartition des patients selon le type de traitement reçu

	Effectif	Pourcentage (%)
Chirurgie radicale + chimiothérapie	14	45,16
Chirurgie radicale seule	10	32,26
Chirurgie palliative seule	5	16,13
Chimiothérapie seule	2	6,45
**Total**	31	100,00

**Aspects évolutifs:** En fonction de l’évolution selon le type de cancer, les patients étaient répartis de la façon suivante (Tableau 6). Le cas de récidive était un néphroblastome et était en rapport avec un suivi irrégulier de la chimiothérapie du à la non disponibilité des drogues de chimiothérapie et à l'impossibilité pour les parents de s'en procurer à l’étranger.

**Tableau 6:** Répartition des patients selon l’évolution en fonction du type de cancer

	Décès	Survie	Récidive	Effectif
Tumeurs des tissus mous	7	8	1	16
Tumeurs germinales	3	5	0	8
Tumeurs osseuses	7	0	0	7
**Total (**%**)**	17 (54,84%)	13 (41,94%)	1 (3,22%)	31 (100,00%)

## Discussion

Les cancers de l'enfant sont rares. En France, la fréquence du cancer de l'enfant est d'environ 1% par rapport à l'ensemble des cancers et les tumeurs solides malignes occupent 70% des cancers de l'enfant [4]. Icher et coll. [5] avaient estimé la fréquence du cancer solide maligne néonatale à 1,2% de l'ensemble des cancers à cet âge. Au Togo, la fréquence des cancers de l'enfant en milieu chirurgical pédiatrique était de 4% par rapport aux cancers de l'enfant et de 0,4% par rapport à tous les cancers avec une incidence d'environ 1cas par an. Le bas âge semblerait être un facteur de risque dans la survenue du cancer chez l'enfant [5, 6–8]. Plus du tiers (40%) des cancers surviennent avant l’âge de 5ans [8]. Bergeron C et Philip T dans leur étude ont relevé que la moitié des enfants atteints d'un cancer l’étaient avant l’âge de 5 ans [7]. Cependant dans notre étude, les patients étaient presque également répartis dans les différentes tranches d’âge (tableau I). Le cancer survient avec une prédominance chez le garçon [6, 9, 10].

Les circonstances de découverte sont fonction du type et du siège du cancer (tableau III). Dans notre étude, les tumeurs des tissus mous ont représenté plus de la moitié des cas puis venaient les tumeurs osseuses (25,80%) et les tumeurs germinales (22,59%). Auvrignon-Decubber A [11] a trouvé que les tumeurs des tissus mous représentaient 14% des cas, les tumeurs osseuses 7% et les tumeurs germinales 2% des cas. Parmi les tumeurs osseuses, le sarcome d'Ewing a représenté environ la moitié des tumeurs osseuses dans notre étude (tableau IV). Ce même constat a été fait par Parkin et coll. [12]. Pour ces auteurs, le sarcome d'Ewing a occupé 50% des tumeurs osseuses. Dans notre étude, le tératome malin était le plus fréquent avec plus de la moitié des tumeurs germinales alors que dans la série de Hadley et coll. [13], c'est plutôt le carcinome embryonnaire qui était le plus fréquent avec 72% des tumeurs germinales. Le néphroblastome était le plus fréquent des tumeurs des tissus mous de notre série (tableau IV). Le même constat a été fait par Campbell et coll. [14] et Isaac et coll. [15] qui ont trouvé que le néphroblastome a occupé respectivement 62% et 58% des tumeurs des tissus mous.

Dans notre étude, aucune tumeur cérébrale n'a été retrouvée. Pour Parkin et coll. [12], les tumeurs malignes cérébrales sont les tumeurs solides les plus fréquentes de l'enfant et représentent environ 25% des néoplasies dans les pays industrialisés. Pour Clough-Gorr K et Von der Weid N [4], les tumeurs malignes cérébrales et le neuroblastome sont nettement plus rares dans les pays en développement mais il est possible que cette différence soit plutôt la conséquence d'un sous diagnostic ou d'un sous enregistrement plutôt que le reflet d'un risque réellement réduit dans ces régions. Le cancer de l'enfant pose de véritables problèmes dans notre contexte de pays en développement.

En effet dans les pays en développement comme le notre, l'ignorance et la négligence des parents à se présenter à l'hôpital dès les premiers symptômes d'une part, le recours aux tradithérapeutes et à l'automédication d'autres parts expliquent les longs délais d’évolution avant la consultation. Dans notre étude, ce délai d’évolution avant de consultation était très long; 4,6 mois en moyenne avec des extrêmes de 2 mois et 14 mois (tableau II). Cependant dans les pays développés, les patients sont vus plus tôt en consultation. Ainsi dans la série de Icher et coll. [5], 63% des patients avaient consulté avant 8 jours. Les pays en développement sont également caractérisés par des moyens diagnostiques très limités de ces tumeurs se résumant dans la majorité des cas à une radiographie standard ou à une échographie comme dans notre série. En effet, ces examens d'imagerie médicale ne permettent pas un diagnostic précoce des tumeurs solides malignes chez l'enfant. Sur le plan thérapeutique, la prise en charge d'un enfant atteint de cancer nécessite une concertation étroite entre différents spécialistes: pédiatre, chirurgien pédiatre, radiologue, cytologiste et anatomopathologiste [16]. Au Togo, l'absence des cette organisation fait que la prise en charge de certains cancers solides de l'enfant est faite exclusivement par l’équipe de chirurgie pédiatrique. Cette prise en charge est parfois très insuffisante en raison du stade tardif de la maladie, du plateau technique et de la non disponibilité de certains moyens thérapeutique (tableau V). Par ailleurs, il n'existe par une franche collaboration entre chirurgien pédiatre et radiologue et anatomopathologiste. Les résultats des examens anatomopathologiques mettent des mois voire des années pour être disponibles en raison du nombre limité des anatomopathologistes. Tous ces éléments handicapent la prise en charge des enfants atteints de cancer et entrainent des répercussions sur les résultats de la prise en charge de ces cancers. Dans notre étude, le taux de décès était de 54,84%. Rubie et coll. [16] dans la prise en charge globale de l'enfant atteint de cancer ont enregistré un taux de survie de 90% contre 0% de décès. Le faible taux de survie dans notre étude s'explique par le diagnostic tardif et la limitation de l'arsenal thérapeutique (absence de radiothérapie et drogues antimitotique non disponibles en permanence). Cependant, le taux de survie est fonction du type de cancer (tableau VI). Ce taux était de 72% et de 95% chez des enfants atteints respectivement de tumeurs germinales et de tumeurs des tissus mous dans la série de Rubie et coll. [16]. Dans notre étude, plus de la moitié des enfants atteints de tumeurs des tissus mous avaient survécu, moins de la moitié de ceux atteints de tumeurs germinales avaient survécu et tous les patients atteint de tumeurs osseuse étaient décédés (tableau VI). Ces résultats pourraient s'expliquer par le fait de les tumeurs osseuses sont hautement métastatique et leur diagnostic dans notre contexte est toujours tardif.

## Conclusion

Les tumeurs solides malignes de l'enfant sont rares chez nous. Elle touche de manière égale toutes les tranches d’âge. Les tumeurs des tissus mous sont les plus fréquentes avec une prédominance du néphroblastome. Les tumeurs solides de l'enfant posent de véritables problèmes en rapport avec le retard diagnostic, les moyens diagnostics et de prise en charge limités. Ces problèmes sont à l'origine du taux très élevé de décès est dans notre contexte de pays en développement.
